# Supplementary material for: Enhancing analogy-based software cost estimation using Grey Wolf Optimization algorithm
Source: PeerJ Comput Sci. 2025 Jun 18;11:e2794. doi: 10.7717/peerj-cs.2794 (PMC12190706; doi:10.7717/peerj-cs.2794)
Supplement: Supplemental Information 7 [file peerj-cs-11-2794-s007.docx]

# Overview

This repository contains the source code and data that we used to perform the experiment in the paper titled "Enhancing Analogy-based Software Cost Estimation using Gray Wolf Optimization Algorithm".

## Repository Structure

The repository is organized into the following directories and files:

### `primary/`

- **Manuscript**

- `cs-102446-Manuscript.docx`: The main manuscript of the article.

- **Figures**

- `cs-102446-Figure1.png` to `cs-102446-Figure15.png`: High-resolution images of the figures used in the article.

- **Tables**

- `cs-102446-Table_1.docx` to `cs-102446-Table_7.docx`: Word documents containing the tables included in the article.

### `supplemental/`

Contains additional supplemental materials related to the study. (Add specific details if available)

## Instructions for Use

1. Manuscript: Open `cs-102446-Manuscript.docx` to view the full text of the article.

2. Figures: The figures used in the manuscript are provided as PNG files. Each figure is named sequentially (Figure1 to Figure15).

3. Tables: The tables included in the article are provided as individual Word documents, named sequentially (Table_1 to Table_7).
